# Classifying biogeographic realms of the endemic fauna in the Afro‐Arabian region

**DOI:** 10.1002/ece3.6562

**Published:** 2020-07-20

**Authors:** Alaaeldin Soultan, Martin Wikelski, Kamran Safi

**Affiliations:** ^1^ Department of Migration Max Planck Institute of Animal Behavior Radolfzell Germany; ^2^ Department of Biology University of Konstanz Konstanz Germany; ^3^ Present address: Department of Ecology Swedish University of Agricultural Sciences Uppsala Sweden

**Keywords:** Afro‐Arabian region, biogeography, cluster analysis, endemic species, indicator species, species diversity patterns

## Abstract

**Aim:**

Understanding diversity patterns and identifying the environmental factors that shape these patterns are essential for ecology and conservation. The Afro‐Arabian region comprises one of the most important biogeographic areas connecting continents. Yet, little emphasis has been put on understanding its endemic fauna in relation to its biogeographic realms. Our objective is to fill the gaps in knowledge on diversity patterns and biogeography that are essential for prioritizing the overdue conservation efforts.

**Location:**

The study area covers mostly the hot desert climate region in North Africa and Arabia, and includes the Mediterranean, Sahel, and Ethiopian highlands (hereafter “Afro‐Arabian region”).

**Methods:**

We used distribution maps developed by IUCN and BirdLife for species endemic to the Afro‐Arabian region belonging to the four tetrapod classes, amphibians, reptiles, birds, and mammals, to identify the endemic richness hotspots. We then used multivariate analyses to delineate biogeographic regions and evaluate their relationship with the environmental factors.

**Results:**

Our study reveals a complex map of the richness hotspots for the endemic tetrapod classes. The main hotspots of endemism were concentrated at the margins of the study area, along the Mediterranean coast, Ethiopian highlands, and along the Red Sea Mountains. We propose classifying the Afro‐Arabian region into three discrete biogeographic realms for endemic amphibians, four for reptiles and birds, and five discrete biogeographic realms for endemic mammals. The identified realms are defined by their environmental conditions and the historical geological processes.

**Main conclusions:**

Richness hotspots of endemic tetrapod classes were heterogeneously distributed in the Afro‐Arabian region. Our results support the hypothesis that species diversity patterns and endemism have been shaped by the environmental conditions and the paleogeographic processes. Each of the identified bioregions is associated with a characteristic set of tetrapod species. Our results are a benchmark for assessing the effectiveness of the protected areas and for implementing conservation plans for biodiversity.

## Graphical abstract

Although the Afro‐Arabian region comprises one of the most important biogeographic areas connecting continents, little emphasis has been paid to understand the classification of its endemic fauna into biogeographic realms. Here, we fill the gaps in knowledge on diversity patterns and biogeography essential for prioritizing conservation efforts that are overdue. We propose classifying the Afro‐Arabian region into three discrete biogeographic realms for endemic amphibian, four for reptiles and birds, and five discrete biogeographic realms for endemic mammals.

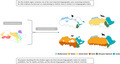

## 1 INTRODUCTION

The largest warm desert—the Sahara and the Arabian Deserts—covers about 17% of the total landmass and harbors about one‐quarter of the terrestrial vertebrate fauna, many endemic and uniquely adapted to harsh environmental conditions (Brito & Pleguezuelos, 2020; Davies et al., 2012; Durant et al., 2012, 2014; Mace, Masundire, & Baillie, 2005; Soultan, 2018). Yet, the desert has always been neglected receiving less attention, particularly from the conservation communities (Brito & Pleguezuelos, 2020; Durant et al., 2012, 2014; Soultan, Wikelski, & Safi, 2019). The desert biodiversity harbors the physiological and genetic basis of species tolerance to extreme temperatures and water stress, which, in turn, could improve our understanding of adaptation to global change (Brito & Pleguezuelos, 2020; Durant et al., 2012).

The biodiversity patterns of the desert have been fundamentally shaped by the long‐term connectivity between Africa and Asia through the *Bab‐el‐Mandeb* strait and Sinai, which allowed the faunal interchange (Ficetola, Bonardi, Sindaco, & Padoa‐Schioppa, 2013; Metallinou et al., 2012, 2015; Šmíd et al., 2013; Winney et al., 2004). Furthermore, the neighboring regions (e.g., the Mediterranean, Sahel, and Ethiopian highlands) have also defined the species composition and endemism of the Sahara and the Arabian Desert through the “Nearest Neighbor Effect” (Patiny & Michez, 2007). Therefore, biodiversity assessment and conservation efforts for the desert require considering the Sahara, the Arabian Desert, and their adjacent regions as one contiguous region (henceforth referred to as the “Afro‐Arabian region”).

Biogeographic regionalization analysis has been successfully applied to identify biodiversity patterns and is considered a promising approach for prioritizing the conservation efforts (Brito et al., 2016; Kreft & Jetz, 2010; Vale et al., 2016). Biogeographic regionalization groups geographic regions into meaningful clusters (henceforth referred to as the “bioregion”) based on the dissimilarity of the species assemblages (González‐Orozco, Laffan, Knerr, & Miller, 2013; González‐Orozco, Thornhill, Thornhill, Knerr, Laffan, & Miller, 2014; Rodrigues, Fig ueira, Vaz Pinto, Araújo, & Beja, 2015). Bioregions can yield important insights into the factors influencing the geographic distribution of species assemblages (Divíšek, Storch, Zelený, & Culek, 2016) beyond being an effective application of conservation practices.

Most often, bioregionalizations have been developed at broad spatial scales, global or continental, and/or for high taxonomic levels, genus, or above (El‐Hawagry & Gilbert, 2014; Kreft & Jetz, 2010; Wallace, 1876). Bioregions identified at broad spatial scales most often neglect sub‐bioregions and areas of species endemism (Linder et al., 2012; Rodrigues et al., 2015). Therefore, accurate identification of these sub‐bioregions requires conducting bioregion analysis at smaller spatial scales (Rodrigues et al., 2015). Bioregions identified at smaller spatial scale allow to (a) validate other bioregions identified at broader spatial scales, and (b) ensure the identification of important conservation areas that might not be identified at broad scales (Brown, Cameron, Yoder, Vences, & Jarvis, 2014; Rodrigues et al., 2015).

Very few studies have attempted to identify the bioregions of the warm desert (specifically at small spatial scale); however, these studies were focused either on a small region (Delany, 1989; Vale et al., 2016) or limited to particular taxa (Cowie, 1989; Ficetola, Falaschi, Bonardi, Padoa‐Schioppa, & Sindaco, 2018; Patiny & Michez, 2007). Brito et al. (2016) carried out a comprehensive analysis to identify the bioregions for the Sahara–Sahel region using endemic and nonendemic species. However, their work was limited to Africa and excluded the Arabian Desert and the adjacent regions. In their study, Brito et al. (2016) proposed five bioregions for endemic species and found an association between these bioregions and the climatic conditions.

In this study, we analyzed the distribution data for the endemic tetrapod species to perform a comprehensive evaluation of bioregions and diversity patterns of the Sahara and the Arabian Desert and their adjacent regions. Our aims were (a) to identify the richness hotspots for endemic tetrapod classes, amphibians, reptiles, birds, and mammals, in the Afro‐Arabian region; (b) to identify the bioregions for each class; (c) to identify a set of indicator species for each bioregion; and (d) to assess the relationships of the bioregions and the environment.

## 2 METHOD

### 2.1 Study area

**Figure 1:**
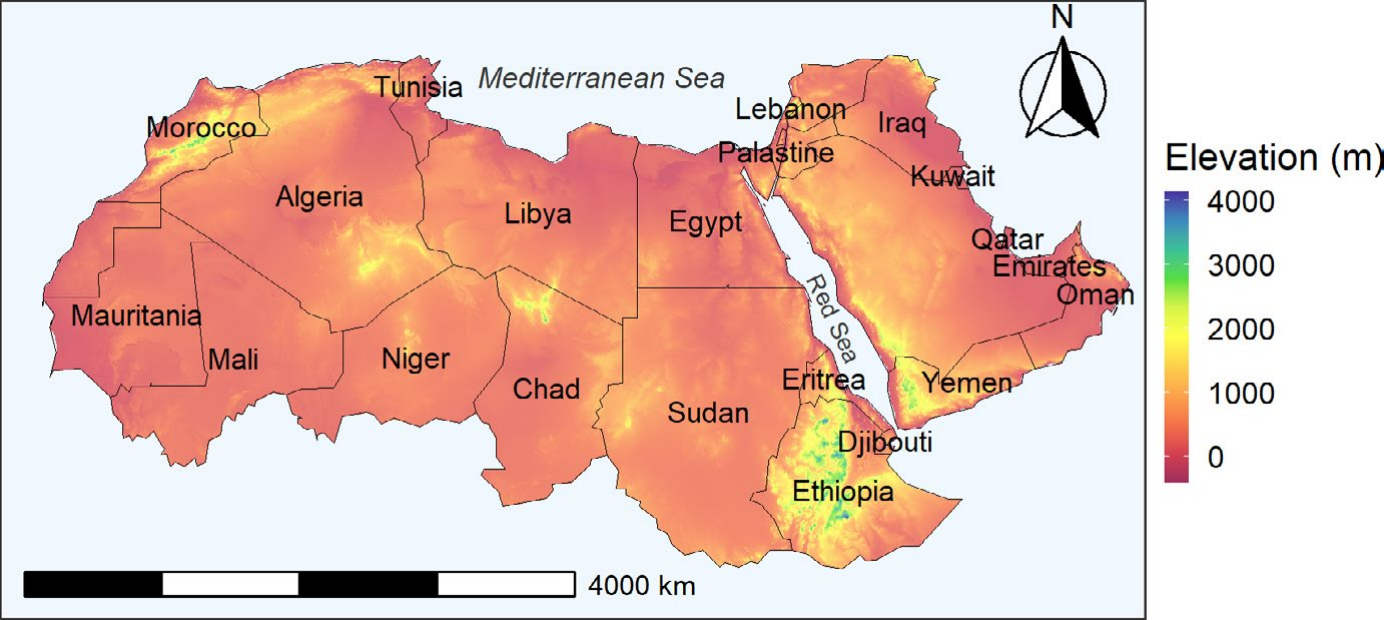
Geographic location of the Afro‐Arabian region overlaid with the political boundaries of the countries. The base map represents the elevation gradient

Our study area, the Afro‐Arabian region, covers the desert in North Africa and the Arabian Desert, and their adjacent regions, the Mediterranean, Sahel, and Ethiopian highlands. The Afro‐Arabian region emerges from the Mediterranean Sea coast in the North and is limited by the political borders of the Sahel countries in the South, and extends from the Persian Gulf westward to the Atlantic Ocean (Figure 1). The Afro‐Arabian region encompasses mainly three distinct biomes, Deserts & Xeric Shrublands, Mediterranean Forests, Woodlands & Scrub, and Tropical & Subtropical Grasslands (Dinerstein et al., 2017).

### 2.2 Species data

We based our analysis on four tetrapod classes (amphibians, reptiles, birds, and mammals) included in the IUCN red list of threatened species and BirdLife (BirdLife International & Handbook of the Birds of the World, 2016; IUCN, 2017) endemic to the Afro‐Arabian region. We considered a species endemic when >70% of its native distribution range was enclosed in the Afro‐Arabian region (Vale & Brito, 2015). We retrieved the digitized geographic range polygons for amphibians, reptiles, and mammals from IUCN (IUCN, 2017), and for birds from BirdLife (BirdLife International & Handbook of the Birds of the World, 2016). These spatial polygons represent the extent of occurrence of the species (Montesino Pouzols et al., 2014; Roll et al., 2017). To fill the gap in reptile distribution, we updated reptile range polygons with additional distributional data from atlases and literature (Baha El Din, 2006; Bar & Haimovitch, 2012; Gonçalves et al., 2018; Metallinou et al., 2012; Sindaco, Jeremčenko, Venchi, & Grieco, 2008, 2013). Overall, 504 species, 51 amphibians, 225 reptiles, 116 birds, and 112 mammals were included in this study (Appendix S1). All species polygons were gridded in 1° resolution grid cells (≈110*110 km). A grid cell that intersected, fully or partially, with a species polygon, was considered as occupied. We used these maps to generate four binary site‐by‐species matrices (i.e., a single matrix for each tetrapod class). Cells with very few species can potentially influence the analysis (Kreft & Jetz, 2010; Yusefi, Safi, & Brito, 2019); therefore, we excluded all grid cells containing fewer than five species.

We calculated species richness for each class by summing up the respective species presence–absence raster maps (Sow, Martínez‐Freiría, Dieng, Fahd, & Brito, 2014; Zhang, Slik, & Ma, 2016). This approach provides a robust estimation for species richness that is less sensitive to spatial scale and incomplete species sampling compared to the traditional approach that estimates the richness only from the recorded species occurrences (Brown et al., 2014).

### 2.3 Delineation of bioregions

We developed the biogeographic regionalization for the Afro‐Arabian region by applying an unweighted pair‐group clustering algorithm based on the arithmetic averages (UPGMA) to the *β*_sim_ dissimilarity matrix. UPGMA is frequently favoured over other clustering algorithms as it minimizes the distortion of the values and avoids emphasizing outliers by including them in their closest groups (Hattab et al., 2015; Kreft & Jetz, 2010; Linder et al., 2012; Zhang et al., 2016). *β*_sim_ calculates the dissimilarity matrix based on the presence data (i.e., not considering the shared absence) and is less sensitive to species richness differences; therefore, it is favoured for regions that share few species such as the Sahara (Linder et al., 2012; Zhang et al., 2016). We determined the optimal number of bioregions using a Kelley–Gardner–Sutcliffe penalty function (KGS) (Kelley, Gardner, & Sutcliffe, 1996). KGS uses species pairwise distance matrix to maximize the dissimilarity between the bioregions and the similarity within the bioregions (Hattab et al., 2015; Kelley et al., 1996; Linder et al., 2012). To map a spatial and quantitative representation of each tetrapod class dissimilarity, we followed the approach developed by Moura, Argôlo, and Costa (2017). We first performed nonmetric multidimensional scaling (NMDS) using the “vegan” package (Jari Oksanen et al., 2017), and then we built an RGB raster using the interpolated NMDS scores, plotted it using “plotRGB” function of the “raster” package (Hijmans, 2016). We used the R script provided by Moura et al. (2017) to interpolate NMDS scores.

### 2.4 Indicator species

To identify the species composition characterizing each bioregion, we calculated the indicator value of a species (*IndVal*) (De Cáceres, Legendre, & Moretti, 2010). *IndVal* takes into account two components: (a) *Specificity*: the relative frequency of each species in a given bioregion, divided by the sum of relative frequencies over all bioregions, and (b) *Fidelity*: the relative frequency of the species within a given bioregion (Hattab et al., 2015; Rodrigues et al., 2015). We calculated the *IndVal* using “multipatt” function implemented in “indicspecies” R package (De Cáceres & Legendre, 2009), where a species is identified as an indicator for a particular bioregion when it's *IndVal* > 0.50 for a *p* < .05 after 999 permutations (Rodrigues et al., 2015).

### 2.5 Bioregion–environment relationships

We considered 10 environmental variables known to define species diversity patterns and distribution. These variables were as follows:

1. Annual mean temperature (Bio1), mean diurnal range (Bio2), and potential evapo‐transpiration (PET), as they have been shown to influence species physiological function such as thermoregulation (Buckley & Jetz, 2007, 2008).
2. Annual precipitation (Bio12), precipitation of the wettest month (Bio13), and distance to water source, as indicators for water availability (Buckley & Jetz, 2007).
3. Net primary production (NPP) as it showed to mediate species coexistence (Buckley & Jetz, 2007, 2008).
4. Altitude, roughness, and soil characteristics, as it showed to shape species dispersal.
5. Climate‐change velocity (hereinafter referred to as “velocity”), as it showed to be strongly associated with endemism and species richness (Loarie et al., 2009; Sandel et al., 2011).

We obtained Bio1, Bio2, Bio12, Bio13, and altitude from WorldClim data (http://www.worldclim.org/), NPP from (https://neo.sci.gsfc.nasa.gov/), PET from (http://www.cgiar‐csi.org/), and soil data from Harmonized World Soil Database (FAO/IIASA/ISRIC/ISSCAS/JRC, 2012). Roughness was calculated from altitude using “terrain” function implemented in “raster” R package (Hijmans, 2016), and the water variable was extracted from land‐cover data available in (http://glcf.umd.edu). We calculated climate‐change velocity over the last 21,000 years using the method developed by Loarie et al. (2009). Based on current mean annual temperature and last glacial maximum mean annual temperature from WorldClim data, we calculated the velocity using “dVoCC” function implemented in “VoCC” R package (García Molinos, Schoeman, Brown, & Burrows, 2019).

We used the relative environmental turnover (RET) method to assess the relationship between environmental variables and the identified bioregions based on species turnover (Buckley & Jetz, 2008; González‐Orozco, Ebach, et al., 2014; Zhang et al., 2016). We used the Gstar hotspot statistic (also known as Getis‐Ord Gi*) to perform the RET analysis (González‐Orozco, Ebach, et al., 2014; Zhang et al., 2016). Gstar with a value >2 or < −2 indicates that the environmental values within a given bioregion are significantly different from expected (González‐Orozco, Ebach, et al., 2014; Zhang et al., 2016). We used the “localG” function in “spdep” R package to calculate Gstar (Bivand, Hauke, & Kossowski, 2013; Bivand & Piras, 2015).

## 3 RESULTS

### 3.1 Species richness

**Figure 2:**
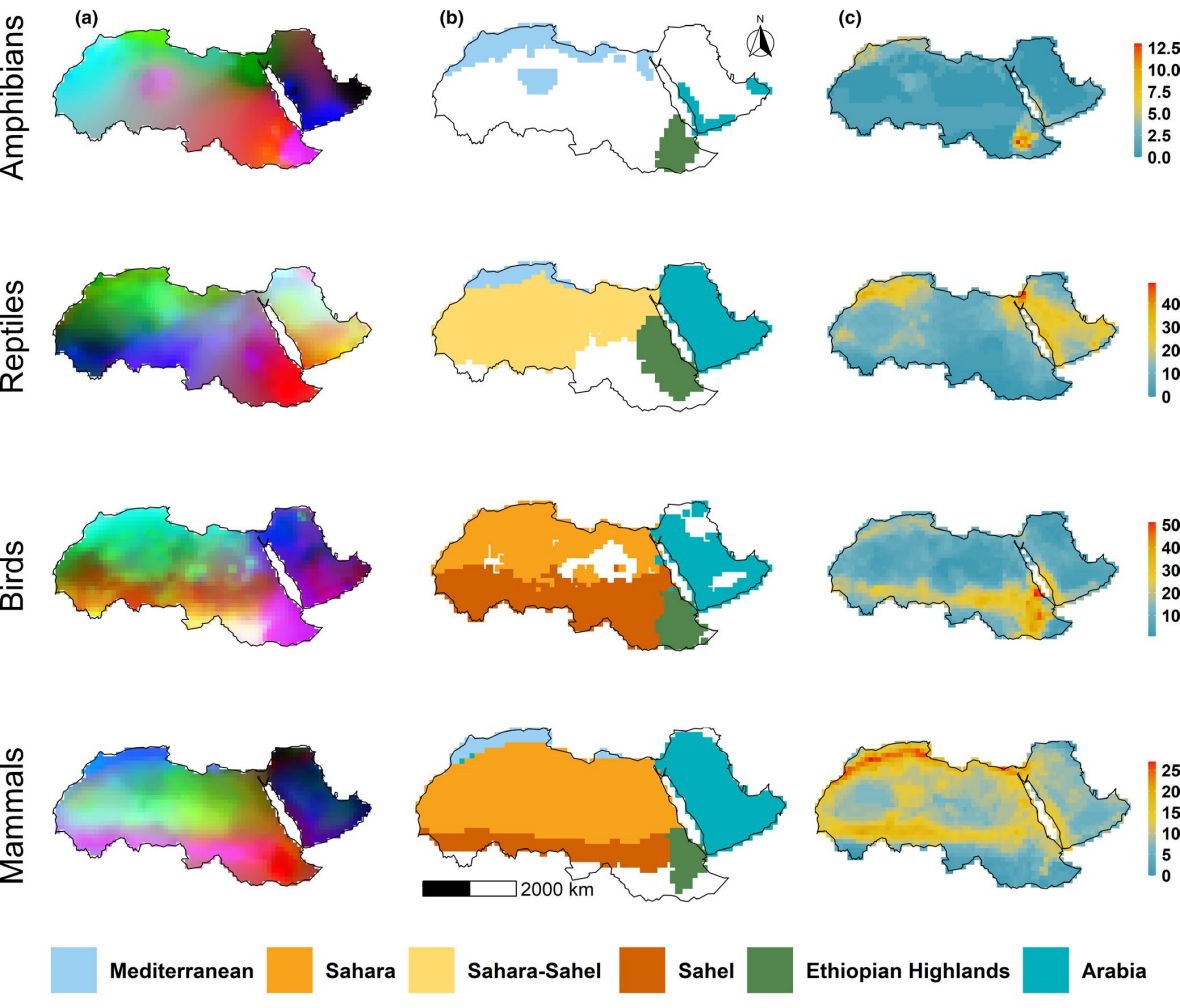
Quantitative representation of species diversity at ≈110*110 km spatial resolution for the endemic tetrapod classes, amphibians, reptiles, birds, and mammals; (a) species turnover as interpolated dissimilarity based on NMDS axes (three axes represented by a RGB scale), (b) the proposed bioregions based on the cluster analysis, and (c) species richness. The scale bars on the right side refer to the score of the species richness, where cold colors represent lower richness and warm colors represent higher richness

Richness hotspots of endemic tetrapod classes were heterogeneously distributed in the Afro‐Arabian region (Figure 2). For endemic amphibians, the hotspot areas are concentrated in the Ethiopian highlands, and at the northwestern edge of the study area. The richness hotspots of endemic reptiles are concentrated in the Levant, the northwestern edge of the study area, and along the Red Sea Mountains in the western edge of the Arabian Desert. For endemic birds, the hotspots are almost all concentrated in the southern part of the study area: the Ethiopian highlands and its adjacent regions, the Sahel and southern part of the Red Sea Mountains. Endemic mammal species richness is high along the Mediterranean Sea coast in North Africa, followed by the Sahel and the Red Sea mountains. Overall, three regions, the Ethiopian highlands, the Mediterranean coast, and the Red Sea Mountains, support high richness.

### 3.2 Bioregions delineation

The biogeographic analysis identified three bioregions for amphibians, four for reptiles and birds, and five bioregions for mammals (Figure 2). For all classes, the Ethiopian region and the Arabian Desert were identified as two distinct bioregions (the Ethiopian Highlands and the Arabia). For amphibians, in addition to these two bioregions, one additional bioregion was identified (the Mediterranean bioregion), which expands over the Mediterranean coast and the northern part of the Sahara. For reptiles, the Sahara and the Sahel regions were grouped into one distinct bioregion (henceforth referred to as “the Sahara–Sahel bioregion”), while the Mediterranean coast at the northwestern edge of the study area was identified as a distinct bioregion (henceforth referred to as “the Mediterranean bioregion”). The identified bioregions for birds and mammals were relatively similar, where both agreed in four bioregions (the “Ethiopian Highlands,” the “Sahara,” the “Sahel,” and the “Arabia”). However, for birds, the “Sahara bioregion” covers the Mediterranean coast and the Sahara, while for mammals, the Sahara and the Mediterranean coast were distinct.

### 3.3 Indicator species

Of the 51 endemic amphibian species analyzed, 17 species had a significant *IndVal* of above 60% and were thus considered indicator species (Appendix S2). For reptiles, a total of 49 species showed significant IndVal of above 50%. Considering the five main bioregions identified for endemic birds, 53 species had a significant *IndVal*, where 35 species were indicators for the Ethiopian Highlands bioregion while only three species for the Sahara bioregion. For mammals, 32 species were considered as indicator species, with 13 species being indicators for the Mediterranean bioregion only, while only four indicator species for the Sahara and four for Arabia.

### 3.4 Bioregions–environment relationship

The results of the Gstar statistic (Table 1) revealed that precipitation is the main driver for amphibian species turnover in the Ethiopian Highlands bioregion, while temperature was the main driver in the Arabia bioregion. The diversity pattern for reptiles was determined mainly by temperature, potential evapo‐transpiration, velocity, and net primary production in the Mediterranean bioregion and by precipitation and soil in the Ethiopian Highlands bioregion. For birds and mammals, the precipitation, velocity, and the topographical features of the habitats were the main drivers of turnover in the Ethiopian Highlands bioregion.

**Table 1:** Gstar statistic values for the environmental variables (column‐wise) corresponding to each identified bioregion for each group

Group	Bioregion	Environmental variables										
		Altitude	Annual precipitation	Precipitation of wettest month	Annual mean temperature	Mean diurnal temp range	Net primary production	Potential evapo‐transpiration	Roughness	Water	Soil	Velocity
Amphibians	Mediterranean	−0.54	−0.58	−0.66	−0.48	0.16	0.50	−0.46	−0.61	0.24	−0.46	0.00
	Arabia	−0.19	−0.78	−0.63	1.58	−1.30	−0.09	0.48	0.38	−0.37	−0.66	1.00
	Ethiopian Highlands	1.74	**2.11**	**2.27**	0.09	0.50	−1.31	0.84	1.47	−0.46	1.62	−0.74
Reptiles	Mediterranean	0.72	1.29	0.41	**−2.74**	−1.67	−1.83	**−2.87**	1.40	−1.11	−0.11	**−2.24**
	Arabia	0.24	−0.17	−0.24	−0.11	−0.89	−0.14	−0.53	0.11	−0.29	−0.34	0.04
	Sahara–Sahel	−0.39	−0.45	−0.34	0.25	0.56	0.46	0.43	−0.46	0.46	−0.39	0.37
	Ethiopian Highlands	1.30	1.99	**2.09**	0.38	0.13	−1.05	0.58	1.40	−1.12	**2.83**	−0.73
Birds	Sahara	−0.21	−0.67	−0.86	−0.92	0.13	0.66	−0.75	−0.07	0.38	−0.46	−0.32
	Arabia	−0.46	0.48	0.77	1.20	0.52	−0.48	1.22	−0.64	−0.15	0.45	0.65
	Sahel	0.42	−0.53	−0.63	−0.33	−1.04	0.26	−0.77	0.37	−0.14	−0.50	0.05
	Ethiopian Highlands	**2.60**	**2.31**	**2.10**	−0.62	−0.42	−1.64	−0.29	**2.68**	−0.77	1.39	−1.72
Mammals	Mediterranean	0.72	1.44	0.36	**−3.10**	**−2.04**	−1.77	**−3.28**	1.83	−1.21	−0.26	**−2.53**
	Arabia	0.29	−0.27	−0.40	−0.29	−1.03	−0.02	−0.73	0.24	−0.30	−0.42	−0.08
	Sahara	−0.30	−0.68	−0.66	0.09	0.55	0.77	0.29	−0.40	0.52	−0.10	0.35
	Sahel	−0.40	1.61	**2.29**	1.40	0.42	−1.98	1.34	−0.62	−0.88	0.74	0.23
	Ethiopian Highlands	**3.35**	**3.83**	**3.29**	−0.75	−0.46	−1.90	−0.42	**3.78**	−1.13	1.65	**−2.05**

Note

Value >2 or < −2 (bold text) means the environmental variable is important for this bioregion.

## 4 DISCUSSION

Our study provides insight into the spatial diversity patterns and bioregion affiliations for endemic tetrapod classes in the Afro‐Arabian regions. We showed that IUCN and BirdLife spatial data with multivariate statistical analysis could identify bioregion affiliations and provide transparent and replicable analyses as a basis for conservation and other ecological studies. The Afro‐Arabian region is characterized by extreme environmental conditions and its remoteness and is thus scarcely investigated. Identifying richness hotspots and delineating the bioregions would be essential for implementing effective conservation plans and conducting the appropriate conservation measures.

In contrast to the previous bioregion analyses (Brito et al., 2016; Vale & Brito, 2015), we considered the impact of the adjacent areas, the sub‐Saharan and the Arabian Desert. This impact, known as “Nearest Neighbor Effect” (Patiny & Michez, 2007), influences species composition and endemism in the Sahara (Patiny & Michez, 2007). Additionally, we carried out a separate analysis for each tetrapod class, whereas the previous studies gathered all groups in one analysis.

Our analysis showed an incongruence among the tetrapod classes, amphibians, reptiles, birds, and mammals, in the richness hotspots, where each class has distinct richness patterns (Figure 2). This finding is in concordance with previous work that showed a heterogeneous distribution for the endemic tetrapod species across the Sahara–Sahel region (Brito et al., 2016). However, other studies conducted at a larger scale (i.e., global and continental) were inconsistent with our results, as they showed a homogeneous richness distribution (Lamoreux et al., 2006; Lewin et al., 2016). Our findings together with previous works (Brito et al., 2016; Rodrigues et al., 2015) confirm that analyses at smaller spatial scales allow for better identification of important conservation areas that might not be identified at broad scales (Brown et al., 2014; Rodrigues et al., 2015). While the richness hotspots occurred on the margins of the Afro‐Arabian region, the center of the study area is also an area of high conservation value because it harbors other endemic species despite the harsh environmental condition. Identifying the endemic richness hotspots should, therefore, be useful for conserving both endemic and nonendemic species. For instance, Lamoreux et al. (2006) demonstrated that prioritizing just 10% of the world's land based on the richness hotspots of only endemic birds would significantly benefit about 60% of world vertebrates. Therefore, prioritizing conservation efforts based on hotspots of endemism would be highly recommended over using the traditional prioritization approach, which relies on richness hotspots of all species.

The amphibians are almost absent from much of the Sahara and the Arabian Desert (Figure 2), which could be because of the extreme environmental conditions. This finding has also been reported in previous studies (Holt et al., 2013; Linder et al., 2012; Vale, Santos, & Brito, 2020), making amphibians obviously poor indicator species for the Sahara. The main three bioregions we identified here for the endemic amphibians (Figure 2), the Mediterranean, Arabia, and the Ethiopian Highlands, are in broad congruence with the updated Wallace bioregions (Holt et al., 2013).

For the endemic reptile species in the Afro‐Arabian regions, we propose four bioregions: Mediterranean, Sahara–Sahel, Ethiopian Highlands, and Arabia (Figure 2). Among the terrestrial vertebrates, reptiles are the only group without a comprehensive analysis (Ficetola et al., 2013; Roll et al., 2017), which limits the possibility to compare our findings with previous results. However, a recent study focusing on the phylogeography of the genus *Acanthodactylus* (Tamar et al., 2016), the most species‐rich genus in the family Lacertidae (≈40 species), combined the Sahara and the Sahel in one bioregion and separated the Sahara from the Arabian Desert. This finding is congruent with our finding (Figure 2) and supports the argument that bioregion analysis can be a useful surrogate of eco‐evolutionary processes in the absence of molecular data (Brito et al., 2016; Carvalho, Brito, Crespo, & Possingham, 2011).

For the endemic bird species in the Afro‐Arabian regions, we propose four bioregions (Figure 2): the Sahara, the Sahel, Arabia, and the Ethiopian Highlands. This result relatively agrees with a previous study that focused only on endemic bird species in the sub‐Saharan (de Klerk, Crowe, Fjeldsa, & Burgess, 2002), where the authors split the Sahel and the Ethiopia regions into two distinct bioregions. These findings and our results are distinct from the bioregions proposed for birds by Holt et al., (2013), where they recognized the Sahara and the Arabian Desert as one bioregion and the Sahel and the Ethiopian montane as another. However, this disagreement is probably an effect of the scale combined with the characteristics of the species. Holt et al. (2013) conducted their study at a global scale. Most often, studies at a global scale exclude species with small ranges to minimize the distortion that could influence the outcome. We, however, restricted our analysis to the endemic species, which are varying in their response to the micro‐environmental conditions, due to local adaptation, which might not be reflected in the global scale analysis and when using all species, including both endemic and nonendemic species. Nevertheless, our analyses agree with Holt et al., (2013), in grouping the Saharan and Mediterranean regions into one bioregion, which we refer to as the Sahara for sake of simplicity.

We proposed five bioregions (Figure 2), the Mediterranean, the Sahara, Arabia, the Sahel, and the Ethiopian Highlands, for endemic mammal species in the Afro‐Arabian regions. These bioregions showed a large degree of congruence with the bioregions proposed in the previous studies (Holt et al., 2013; Kreft & Jetz, 2010), particularly the Mediterranean and the Sahel bioregions. However, we, together with Kreft and Jetz (2010), disagree with Holt et al., (2013) in grouping the Sahara and the Arabian Desert in one distinct bioregion (the Saharo‐Arabia).

Our analysis showed that the patterns of species turnover were relatively similar among the tetrapod classes, particularly within the Arabian Desert and the Sahara (Figure 2). This finding is consistent with recent work that found a similarity between the turnover patterns of endemic reptiles and mammals within the Sahara (Vale et al., 2020). Our analysis showed that species turnover patterns are influenced by environmental conditions (Table 1). The impact of the environmental conditions, however, varied among bioregions and classes. In line with our findings, previous studies (González‐Orozco et al., 2013; Hattab et al., 2015; Zhang et al., 2016) also have reported the importance of the environmental variables on the patterns of species turnover. For instance, we found that species turnover in the Mediterranean bioregion is shaped mainly by the temperature and the potential evapo‐transpiration. These variables are closely linked to the water–temperature balance, where, according to the water–temperature balance hypothesis, the water and energy jointly constrain species distribution patterns (Buckley & Jetz, 2007, 2008). In general, the most important environmental factors were precipitation and temperature, followed by the topographical variables. However, the impact of these factors on the patterns of species turnover in the central region of the study area, which includes three bioregions, the Sahara, Arabia, and the Sahel, was not as strong as in the other bioregions. This could be a result of the huge area size of these three bioregions, the Sahara, Arabia, and the Sahel, in comparison with other bioregions. Additionally, species in these three bioregions are characterized by relatively wide ranges, which, in turn, dilute the impact of the environmental factors. The relative environmental turnover analysis suggests that species diversity patterns in these three bioregions are shaped by the geological conditions rather than environment conditions (Table 1).

Our analysis shows that three regions, the Ethiopian highlands, the Mediterranean coast, and the Red Sea Mountains, support high richness (Figure 2). This could be a result of including the Mediterranean and the Ethiopian highlands in our study. These regions are home to many species with small ranges. Although the Mediterranean and the Ethiopian highlands are distinct bioregions (Dinerstein et al., 2017), including them in the analysis was important to account for the “Nearest Neighbor Effect” (Patiny & Michez, 2007). This effect has fundamentally shaped species composition and endemism in the study area and allowed for faunal interchange between the continents (Metallinou et al., 2012; Winney et al., 2004). This interchange resulted in (a) species with wide distribution over Africa and Asia (e.g., *Panthera pardus*), (b) very closely related species with allopatric distributions, for instance, *Gazella dorcas, Acomys cahirinus*, and *Oryx dammah* in Africa, and *Gazella saudiya, Acomys dimidiauts*, and *Oryx leucoryx* in Asia, and (c) species with disjunct distribution ranges between Africa and Asia, for instance, *Papio hamadryas* distributed in the Horn of Africa and southwestern of the Arabian peninsula (Metallinou et al., 2012, 2015; Newman, Jolly, & Rogers, 2004; Winney et al., 2004).

The identified bioregions harbor unique species that are highly adapted to the environmental conditions in these bioregions (Appendix S2). These indicator species can be used to evaluate the success of conservation management in these bioregions. For instance, the North African Water Frog (*Pelophylax saharicus*), Checkerboard Worm Lizard (*Trogonophis wiegmanni*), and Maghreb Garden Dormouse (*Eliomys munbyanus*) are among the identified indicator amphibians, reptiles, and mammals, respectively, that inhabit the Mediterranean bioregion (Appendix S2). The North African Water Frog exhibits high ecological plasticity (Marisa Esteban, García‐París, Buckley, & Castanet, 1999). For instance, it can survive the summer period without having to go through estivation (Escoriza & Hassine, 2019; Marisa Esteban et al., 1999). Moreover, this frog can extend its breeding season; meanwhile, the adult females can have oocytes of different maturity ages (Marisa Esteban et al., 1999). The other species, the Checkerboard Worm Lizard and Maghreb Garden Dormouse, are known to be highly adapted to the arid and semi‐arid environment (Barata et al., 2011; López‐García, Agustí, & Aouraghe, 2013). A previous paleontological study showed that the key mammals in the Mediterranean bioregion adapted to dry condition since the Holocene period which was slightly drier than today (López‐García et al., 2013).

The Dhofar Toad (*Duttaphrynus dhufarensis*), Arnold's Fringe‐fingered Lizard (*Acanthodactylus opheodurus*), Arabian Babbler (*Argya squamiceps*), and Cheesman's Gerbil (*Gerbillus cheesmani*) are among the indicator species (amphibians, reptiles, birds, and mammals, respectively), that exhibit high adaptability to the Arabia bioregion (Appendix S2). For instance, Dhofar Toad is reported in dry habitats and in regions where no water bodies are present (Soorae, Els, Gardner, & Alqamy, 2013). Dhofar Toad goes through long estivation, more than two years, to avoid a drought period (Balletto, Cherchi, & Gasperetti, 1985; Soorae et al., 2013). The other indicator species are characterized by high ecological plasticity that allows these species to survive the harsh environmental conditions. For instance, they can change their activity time during the day and change their food habit according to the available resources and their physiological demands (Collar & Robson, 2020; Scott & Dunstone, 2000).

The indicator species for the Sahara and the Sahel bioregions are characterized by relatively wide distribution ranges compared to the indicators of the other bioregions. For instance, the ranges of the Long Fringe‐fingered Lizard (*Acanthodactylus longipes*), African Houbara (*Chlamydotis undulata*), and Sudan Gerbil (*Gerbillus nancillus*) extend from the Red Sea westward to the Atlantic coast (Baha El Din, 1996; Collar & Garcia, 2020; Happold, 2013). The lizard and gerbil species use the vegetation as shelters and dig burrows to avoid the high temperature (Baha El Din, 2006; Sindaco et al., 2008). The indicator species of Ethiopian highland bioregion (e.g., Eritrea Clawed Frogs (*Xenopus clivii*), Blanford's Blind‐snake (*Afrotyphlops blanfordii*), Black‐billed Woodhoopoe (*Phoeniculus somaliensis*), and Ethiopian Genet (*Genetta abyssinica*)) are characterized by a confined latitudinal range and broad altitudinal ecological range (Broadley & Wallach, 2009; Evans, Bliss, Mendel, & Tinsley, 2011; Ferguson, Roble, & McDonough, 2019; Ligon & Kirwan, 2020; Yalden, Largen, Kock, & Hillman, 1996).

Prioritizing conservation efforts requires detailed information about diversity patterns and their relationship with the environmental conditions (Ficetola et al., 2013; Rondinini, Wilson, Boitani, Grantham, & Possingham, 2006; Whittaker et al., 2005). Such information is not always available, particularly in regions with extreme environmental conditions, and it is necessary to fill this gap prior to any conservation action (Ficetola et al., 2013). Our study provides the required information about species diversity patterns and bioregion affiliations to implement an effective conservation plan in the Afro‐Arabian region. Species richness and turnover have useful applications in conservation, as they could be used to assess efficiency of the already established network of protected areas and guide the future improvements (Lasram, Hattab, Halouani, Romdhane, & Le Loc'h, 2015). Lamoreux et al. (2006) showed that prioritizing just 10% of the world's land based on the richness hotspots of endemic bird species included about 60% of all endemic vertebrates and a large number of nonendemic species. Therefore, using the identified endemic richness to prioritize conservation efforts is a useful approach allows for conserving both endemic and nonendemic species. The findings of the current study (distinct bioregions and its indicator species) can be used to parametrize spatial zonation models in order to achieve the optimal conservation practice that maximizes the benefits while minimizing the cost and effort.

## 5 CONCLUSION

This study contributes toward delineating bioregions and the diversity patterns of the endemic tetrapod classes in the Afro‐Arabian region. It reveals the distinct species richness patterns and the hotspot areas of endemism. Our results support the hypothesis that species diversity patterns and endemism have been shaped by the environmental conditions and the paleogeographic processes. The identification of distinct bioregions for each tetrapod class allows prioritizing the conservation efforts in the Afro‐Arabian region. Further analysis is required to identify the threatened species and the underlying processes leading to species extinction, which would contribute greatly toward the urgently needed conservation prioritization efforts in the Afro‐Arabian region.

## CONFLICT OF INTEREST

The authors declare no conflict of interest.

## AUTHOR CONTRIBUTION

**Alaaeldein Soultan:** Conceptualization (equal); Data curation (equal); Formal analysis (equal); Methodology (equal); Writing‐original draft (equal); Writing‐review & editing (equal). **Martin Wikelski:** Conceptualization (equal); Funding acquisition (supporting); Investigation (lead); Project administration (supporting); Resources (supporting); Supervision (lead); Writing‐review & editing (equal). **Kamran Safi:** Conceptualization (equal); Formal analysis (supporting); Funding acquisition (supporting); Investigation (lead); Methodology (supporting); Project administration (lead); Resources (lead); Supervision (lead); Writing‐original draft (equal); Writing‐review & editing (equal).

## Supporting information

AppendixS1

AppendixS2

## Data Availability

A list of all species considered in this study is presented in Appendix S1. The digitized geographic range polygons for mammals, amphibians, and reptiles are available at IUCN website (https://www.iucnredlist.org/resources/spatial‐data‐download), while the digitized geographic range polygons for birds are available at BirdLife website (http://datazone.birdlife.org/species/requestdis).
